# Long-term intensive management reduced the soil quality of a *Carya dabieshanensis* forest

**DOI:** 10.1038/s41598-023-32237-9

**Published:** 2023-03-28

**Authors:** Cheng Huang, Songling Fu, Xiaomin Ma, Xiaoxiang Ma, Xiaoliang Ren, Xinxin Tian, Yinhao Tong, Feiyang Yuan, Hua Liu

**Affiliations:** School of Forestry and Landscape Architecture, Anhui Agricultural University, 130 Changjiang W Rd, Hefei, 230036 Anhui China

**Keywords:** Ecology, Forest ecology, Forestry

## Abstract

The evaluation of soil quality can provide new insights into the sustainable management of forests. This study investigated the effects of three types of forest management intensities (non-management (CK), extensive management (EM), and intensive management (IM)), and five management durations (0, 3, 8, 15, and 20 years) on the soil quality of a *Carya dabieshanensis* forest. Further, minimum data sets (MDS) and optimized minimum data sets (OMDS) were established to evaluate the soil quality index (SQI). A total of 20 soil indicators representing its physical, chemical, and biological properties were measured for the 0–30 cm layer. Using one-way ANOVA and principal component analysis (PCA), the total data set (TDS), the minimum data set (MDS), and optimized minimum data set (OMDS) were established. The MDS and OMDS contained three (alkali hydrolyzed nitrogen (AN), soil microbial biomass nitrogen (SMBN), and pH) and four (total phosphorus (TP), soil organic carbon (SOC), AN, and bulk density (BD)) soil indicators, respectively. The SQI derived from the OMDS and TDS exhibited a stronger correlation (r = 0.94, p < 0.01), which was suitable for evaluating the soil quality of the *C. dabieshanensis* forest. The evaluation results revealed that the soil quality was highest during the early stage of intensive management (IM-3), and the SQI of each soil layer was 0.81 ± 0.13, 0.47 ± 0.11, and 0.38 ± 0.07, respectively. With extended management times, the degree of soil acidification increased, and the nutrient content decreased. Compared with the untreated forest land the soil pH, SOC, and TP decreased by 2.64–6.24%, 29.43–33.04%, and 43.63–47.27%, respectively, following 20 years of management, while the SQI of each soil layer decreased to 0.35 ± 0.09, 0.16 ± 0.02 and 0.12 ± 0.06, respectively. In contrast to extensive management, the soil quality deteriorated more rapidly under longer management and intensive supervision. The OMDS established in this study provides a reference for the assessment of soil quality in *C. dabieshanensis* forests. In addition, it is suggested that the managers of *C. dabieshanensis* forests should implement measures such as increasing the amount of P-rich organic fertilizer and restoring vegetation to increase soil nutrient resources for the gradual restoration of soil quality.

## Introduction

As a critical component of forests and other types of terrestrial ecosystems, soil regulates the decomposition of organic materials and mineral transformation, while maintaining the balance of nutrients between plants and the ambient environment^1^. Various intensities of land degradation have been observed on a global scale as the result of increased population and land management activities^2^. Thus, there is widespread concern as to how the degradation of farmland and forest soils might be reversed to achieve sustainable land management, particularly in developing countries^3,4^.

Anthropogenic commercial production and management activities in terrestrial environments (e.g., forests, farmland, and grasslands) are the most common soil disruptors^5^. Intensive forest management has been extensively implemented worldwide to increase forest productivity, or the generation of forest products (e.g., nuts, rubber, wood, etc.)^6–8^. In China, intensive forest management practices occur primarily in bamboo forests and commercial forest plantations, which involve fertilization, the clearing of understory vegetation, and deep plowing^9,10^, which can increase yields and reap significant economic returns over the short term; however, long-term intensive management may result in land degradation^11,12^. A study of bamboo forests in Eastern China indicated that intensive management significantly reduced the carbon (C), nitrogen (N), and phosphorus (P) contents of rhizospheric soil, while under greater management intensity the conversion of soil C and N was reduced and mineralization increased^9^. Further, high-intensity forest management practices have negative impacts on the soil organic C content and unstable C pool, while microbial communities and enzyme activities are affected to variable degrees, contingent on the intensity level^11,13^. In the southern tropical regions of China, the intensive management of *Hevea brasiliensis* have inflicted serious damage on the soil, and the local economic development and ecosystem balance have been affected^2,14^. To restore soil health and maintain sustainable land use, operators have undertaken a series of measures such as planting crops under monocultured *Carya illinoensis* forests^8^, replacing chemical fertilizers with organic fertilizers in *Camellia oleifera* forests^15^, and returning straw to the field^16^. These strategies can augment soil conditions to a certain extent; however, further improvements necessitate the comprehensive analysis of soil quality to facilitate the implementation of more enhanced procedures.

As an essential parameter for the evaluation of its health status, its quality reflects the capacity to maintain the health of animals, plants, microorganisms^17^. There is no doubt that forest management practices have varying degrees of impact on soil quality; however, there remains a lack of universally applicable tools insofar as the methods and systems employed to evaluate soil quality^18^. The soil quality index (SQI) is a well-recognized evaluation system, wherein the flexible requirements for evaluation parameters have supported it broad use for the assessment of soil quality on a global scale^17,19,20^. As reported in the literature, several representative indicators for the development of a minimum data set (MDS) may be selected primarily through principal component analysis (PCA). The indicator screening and filtration rates can typically reach > 50%, which significantly saves the labor and time required for observations; thus, this lightweight evaluation strategy has been extensively recognized^21–24^. However, due to the complexity and heterogeneity of soil systems, further investigations are often required to evaluate the soil quality of different regions, various vegetation types, and diverse land management techniques^25,26^.

As a subspecies of the genus Hickory, *Carya dabieshanensis* is endemic to the Dabie Mountains of China, where a large area of natural forest was discovered in the 1970s. Due to the rich nutritional value and high-quality taste of its nuts, this tree was propagated across a large region over the last 20 years^27^. However, long-term intensive management has resulted in acidification and nutrient imbalances in the soil of *C. dabieshanensis* forests; thus, the quality of soil health and sustainable productivity are under threat. Moreover, as this species is concentrated in the Dabie Mountains, few researchers have paid attention to the impacts of management on the soil quality of these forest lands, and lacked the tools required to assess soil quality. An elucidation of the impacts of various forest stand management intensities and their duration on the soil quality of *C. dabieshanensis* forests may serve as essential references for their sustainable management. Therefore, for this study we endeavored to further clarify how intensive management drives changes in forest soil quality by observing them in *C. dabieshanensis* forests under different management intensities and durations. Simultaneously, it provided a lightweight tool for soil quality assessment in this area.

This research aimed to examine the shifts in soil physicochemical properties and soil quality of a *C. dabieshanensis* forest under different management intensities and durations. We hypothesized that (1) long-term intensive management reduces the nutrient content and activities of soil enzymes in *C. dabieshanensis* forests; (2) Intensive management is more likely to lead to soil degradation than extensive management; (3) Soil organic carbon (SOC) and soil pH are the primary factors that affect soil quality. The objectives of this paper were to: (1) Identify the key indicators that affect soil quality in *C. dabieshanensis* forests toward the establishment of a MDS; (2) Analyze the effects of management intensity and duration on the soil quality of *C. dabieshanensis* forests; (3) Evaluate what the forest soil quality in the study area was.

## Materials and methods

### Study area

**Figure 1:**
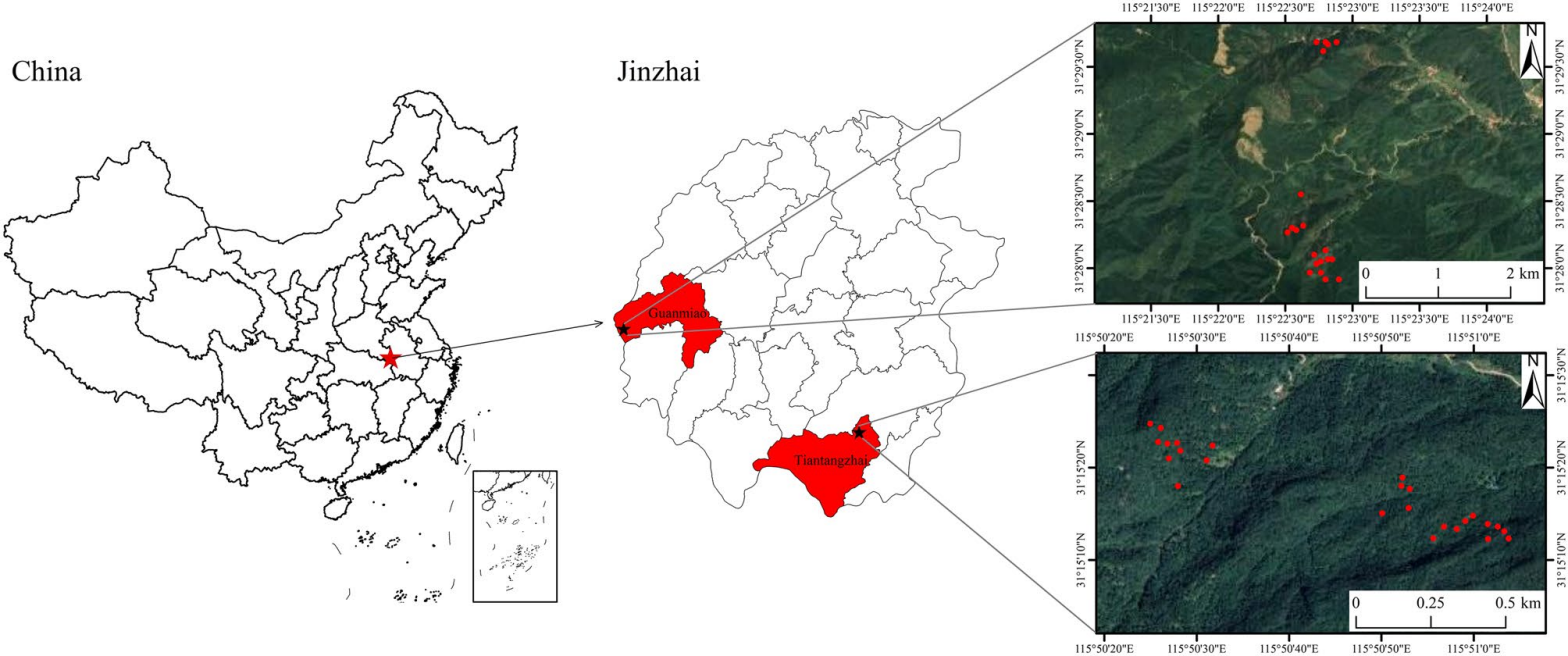
Map of the study area. Note: maps and satellite imagery were generated using the ArcGIS10.8 software from American ESRI Company. https://support.esri.com/en/Products/Desktop/arcgis-desktop/arcmap.

The study site is situated in the Dabie Mountain Forest Area, Tintangzhai Town, and Guanmiao Township, in Jinzhai County, Anhui Province, China. This region is home to a subtropical monsoon humid climate with average temperatures that range from 26 °C in summer to 2 °C in winter, an altitude of 150–1353 m, and average annual precipitation of 1300 mm^28^. The soil thickness ranges from 30 to 100 cm, which is mainly yellow–brown loam (70% medium loam and sandy loam) and slightly acidic (pH 4.5–6.5)^29^ (Fig. 1). Upwards of 90% of the forest area is populated with *C. dabieshanensis*, which contains a small amount of fir, ox nose plug, and maple, with almost no shrub layer under human management. The dominant herbs include *Duchesnea indica*, *Erigeron annuus*, *Aster trinervius*, and *Stellaria chinensis*.

The *C. dabieshanensis* forests under study were natural secondary forests that have been managed since 2000. According to the intensity of management practices, it was divided into three categories (non-management (CK), extensive management (EM), and intensive management (IM)). For the unmanaged model (CK), farmers gathered fruit only in the autumn and performed several other basic management activities. The extensive management (EM) forest involved manual management methods that were simple and extensive in the early stage, which included the removal of almost all trees in the forest, except for *C. dabieshanensis,* to facilitate the collection of fruit. The understory vegetation was removed every August by a lawn mower, with no fertilization. In contrast, intensive management (IM) forest areas were more concentrated, as any trees other than *C. dabieshanensis* were removed from the IM forest area at the beginning of management. A lawn mower was used to clear the understory vegetation twice a year (June and August) and compound fertilizer (N:P_2_O_5_:K_2_O = 13:5:7) (375 kg·hm^−2^) was applied twice (June and August), each year. Organic fertilizer (45% organic matter content) (1500 kg·hm^−2^) was applied once a year from November–December. According to the actual onset of business activities, five time periods were selected as the management time series (0 year (CK), 3 years, 8 years, 15 years, and 20 years).

### Sampling design

Based on the information provided to us by local operators, in June 2022 we divided the forest area by management intensity (CK, EM, and IM), and employed a repeating chronological technique to investigate the forest stands with different years of operation (0 year, 3 years, 8 years, 15 years, and 20 years). We surveyed a total of nine forest areas (CK, EM-3, EM-8, EM-15, EM-20, IM-3, IM-8, IM-15, and IM-20) with five sample plots in each, for a total of 45 sample plots. For each plot, a 20 × 20 m section was randomly established to represent the forest stand, and all plots were set on the north slope (20–30°) of the mountain at > 100 m from the road and farmland. To reduce spatial autocorrelation, the distance between each forest area was > 500 m, and the distance between each plot was > 100 m. The operation data for all forest areas were obtained from the management records of Xiyuan *Carya dabieshanensis* Professional Cooperative and Jinzhai County Tongchuang *Carya dabieshanensis* Development Co., Ltd.

Soil samples were collected from the test site in July 2022. Following the removal of plants and litter from the surface of each plot, soil samples were extracted according to the "S" sampling method using a 100 cm^3^ ring knife. For each tree community a topsoil (0–30 cm) sample was collected, and each was divided into three soil layers (0–10 cm, 11–20 cm, and 21–30 cm). This sampling technique was repeated for each layer, where samples from the same soil layer following the removal of fine root and stones were mixed into new samples, for a total of 135 mixed soil samples were collected. All soil samples were sealed and transferred to the laboratory at low temperature and stored at 4 °C in a freezer pending the detection of soil enzyme activities, as well as physical and chemical properties.

### Laboratory analysis

All sample processing and testing work was completed in a laboratory at Anhui Agricultural University, where the samples were divided into two portions. Fresh soil samples were used for the detection of total protease (T-Pro), sucrase (S-SC), acid phosphatase (S-ACP), β-glucosidase (S-β-GC), soil microbial biomass carbon (SMBC), soil microbial biomass nitrogen (SMBN), and soil microbial biomass phosphorus (SMBP) within 48 h. Used the ring knife method, the remaining portion was used to collect samples to measure the soil bulk density (BD) and soil moisture content (MC), while the determination of total soil porosity (TPO) was achieved by specific gravity^30^. Following air drying, the soil urease (S-UE), pH value, conductivity value (EC), soil organic carbon (SOC), total nitrogen (TN), total phosphorus (TP), total potassium (TK), alkali hydrolyzed nitrogen (AN), available phosphorus (AP), and available potassium (AK) content were quantified. Among them, the BD was found using the ring knife method, whereas a soil pH meter (Mettler Toledo, FE28-Standard, Switzerland) was employed for pH measurements in a soil/water (1:2.5) leachate. The EC value was determined using a conductivity meter (Mettler Toledo, FE38-Standard, Switzerland) at 25 °C using a soil/water (1:5) leachate^24^. The soil TN was measured using an Automatic Kjeldahl nitrogen analyzer (DRICK, DRK-K616K, China). The SOC content was determined by potassium quantified via oxidation-external heating, whereas the AN content was quantified using the alkali-hydrolysis reduction diffusion technique^31^. Further, the SMBC, SMBN, and SMBP were determined via chloroform fumigation extraction and the soil AP and TP were measured using an automatic discontinuous chemical analyzer (DeChem-Tech, Clever Chem Anna, Germany)^32^. The TK and AK were determined using a flame photometer (SHERWOOD, M410, UK). Finally, the detection of T-Pro, S-SC, S-ACP, S-β-GC, and S-UE was done using (ADS-W-D008, ADS-W-TR007-96, ADS-W-TR008, ADS-W-TR003-96, and ADS-W-TR001-96) test kits, which were provided by Jiangsu (China) Aidisheng Biotechnology Co., Ltd.

### Soil quality evaluation

We selected 20 soil indicators (BD, MC, TPO, EC, TN, TP, TK, AN, AP, AK, pH, SOC, SMBC, SMBN, SMBP, T-Pro, S-SC, S-ACP, S-β-GC, and S-UE) as the full data set (TDS) and adopted the membership function method and SQI for comprehensive evaluation. Each group of MDS was filtered using three steps as follows: (1) The TDS indicators were analyzed by principal component analysis (PCA), and the principal components were selected with eigenvalues of ≥ 1. The soil indicators were divided into groups with a load of ≥ 0.5 for each principal component. For metrics that met a load of ≥ 0.5 across multiple principal components, the group with the lowest correlation was entered with other metrics in the same group. (2) The Norm value of each indicator was calculated following grouping, where the larger the Norm value the stronger its capacity to interpret comprehensive data. Indicators with a Norm value in the top 10% range were selected for each group. (3) Correlations between the selected indicators were compared in each group, where if the correlation was strong (p < 0.05), the indicators with the highest Norm value were determined to enter the MDS If the correlations were low (p > 0.05), all were entered into the MDS. The Norm value of the evaluation indicators was calculated as follows:

1 $${\text{N}}_{ik} = \sqrt {\mathop \sum \limits_{i}^{k} \left( {\mu_{ik}^{2} \beta_{k} } \right)}$$

where, N_*ik*_ is the comprehensive load of the *i*-th variable on the top k principal components of the eigenvalue ≥ 1; μ_*ik*_ is the load of the i-th variable on the *k*-th principal component; and β_*k*_ is the eigenvalue of the *k*-th principal component.

Considering that the key factors which affect soil quality in the different soil layers might vary, the screening method was optimized for the inclusion of the MDS indicators. Initially, the dimensionality of the TDS of each soil layer was reduced, after which the minimum data set (MDS) of three soil layers was established. Next, the indicators cross-shared in the MDS of each soil layer (at least being present in the MDS of two soil layers) were selected for the optimized MDS (OMDS).

The standard scoring function was utilized to calculate the metric score. The positive and negative effects of the assessment of soil quality in the study area according to different indicators could be divided into two functional types. When the soil quality level was higher with increased soil indicators, the S-type membership function (Eq. 2) was applied, which was applicable to MC, TPO, TN, TP, TK, AN, AP, AK, SOC, MBC, MBN, MBP, T-Pro, S-SC, S-ACP, S-β-GC, S-UE, and pH (< 7). Conversely, when the soil quality decreased due to higher soil indicator parameters, the selection of the inverse S-type membership function (Eq. 3) was applied, which was applicable to BD and EC.

2 $${\text{N}} = \, \left( {{\text{X}}_{i} - {\text{X}}_{imin} } \right) \, / \, \left( {{\text{X}}_{imax} - {\text{X}}_{imin} } \right)$$

3 $${\text{N}} = {1} - \, \left( {{\text{X}}_{i} - {\text{X}}_{imin} } \right) \, / \, \left( {{\text{X}}_{imax} - {\text{X}}_{imin} } \right)$$

where, X_*i*_ is the measured value of each evaluation indicator, while X_*imax*_ and X_*imin*_ are the maximum and minimum values for each evaluation metric, respectively.

The principal component analysis of MDS was performed to obtain the common factor variance of each MDS indicator. The proportion of the common factor variance of each indicator to the sum of the common factor variance values was calculated, and the weight W of each MDS indicator was obtained. Finally, the membership values and weights of each evaluation indicator were weighted, and the SQI was calculated using the formula:

4 $${\text{SQI}} = \mathop \sum \limits_{i = 1}^{n} W_{i} N_{i}$$

where, SQI represents the SQI, W_*i*_ represents the weight of the *i*-th evaluation indicator, N_*i*_ is the *i*-evaluation indicator membership value, and n is the number of evaluation indicators.

### Data analysis

All data analysis was performed in R 4.1.3. The comparative analyses of soil physicochemical parameters were performed using a one-way ANOVA with the LSD test, where results with p < 0.05 were considered as statistically significant. Pearson correlation coefficients were used to compare the correlations between soil indicators, and principal component analysis (PCA) was employed to screen soil indicators for entry into MDS and OMDS. To understand the explanatory power of MDS and OMDS on the SQI, we used the 'basic trendline' package^33^ to fit the linear regression to quantify the relationship between the MDS and TDS.

## Results

### Influence of management intensity and duration on the characteristics of soil quality evaluation indicators

Management intensity and duration significantly impacted the soil nutrient content and biological activities of *C. dabieshanensis* forests and altered the physical structures of the soil (Table 1). During the initial stage of intensive management (IM-3), the TN, AN, AK, SMBC, urease, sucrase, protease, and EC contents of the soil increased significantly (p < 0.05). The soil moisture content was also highest during this time, where this early stage of extensive management (EM-3) also showed a similar phenomenon to some extent. It was worth noting that the soil pH, TP, and AP decreased to varying degrees following commercial management activities, and other indicators in the soil such as SMBP, SMBN, urease, sucrase TN, AN, TK, AK, and TPO also exhibited a downward trend under longer operational timelines.

**Table 1:** Statistics of soil chemical, physical, and biological indicators in *C. dabieshanensis* forests under different management intensities and durations.

Indicator	CK	IM-3	IM-8	IM-15	IM-20	EM-3	EM-8	EM-15	EM-20
TN (g·kg^−1^)	1.87 ± 0.56a	3.22 ± 0.96d	2.75 ± 0.74c	2.38 ± 0.52abc	2.33 ± 0.64ab	2.42 ± 0.6.3bc	2.03 ± 0.63ab	2.01 ± 0.42ab	1.94 ± 0.51ab
TP (g·kg^−1^)	0.55 ± 0.14de	0.60 ± 0.13 e	0.50 ± 0.12cd	0.36 ± 0.09ab	0.29 ± 0.06a	0.50 ± 0.15cd	0.44 ± 0.14bc	0.36 ± 0.08ab	0.31 ± 0.07a
TK (g·kg^−1^)	23.17 ± 1.72cd	24.25 ± 3.49d	23.07 ± 4.08cd	23.30 ± 2.10cd	22.00 ± 3.05cd	20.84 ± 3.97bc	19.17 ± 2.44ab	18.60 ± 3.38ab	17.53 ± 3.15a
pH	5.29 ± 0.12d	5.20 ± 01.8bc	5.01 ± 0.15a	4.98 ± 0.15a	4.96 ± 0.18a	5.25 ± 0.14cd	5.15 ± 0.10bc	5.13 ± 0.13b	5.15 ± 0.09bc
SOC (g·kg^−1^)	20.25 ± 6.21c	21.90 ± 8.48c	18.27 ± 5.35abc	17.23 ± 4.12abc	13.56 ± 5.10a	20.05 ± 7.06bc	17.54 ± 6.24abc	15.39 ± 4.14ab	14.29 ± 4.41a
AN (mg·kg^−1^)	207.00 ± 53.52a	309.67 ± 90.39c	267.11 ± 69.98bc	227.41 ± 60.59ab	207.88 ± 66.66a	231.63 ± 55.71ab	216.71 ± 75.21ab	207.27 ± 48.49a	195.88 ± 51.60a
AK (mg·kg^−1^)	57.38 ± 11.75b	75.22 ± 17.33c	60.24 ± 15.49b	59.26 ± 13.79b	58.25 ± 15.87b	53.18 ± 7.31ab	51.77 ± 16.82ab	45.62 ± 12.65a	49.20 ± 15.04ab
AP (mg·kg^−1^)	15.91 ± 4.89c	13.73 ± 5.39bc	9.83 ± 4.17a	9.75 ± 4.08a	8.91 ± 3.68a	12.38 ± 5.23ab	11.46 ± 4.72ab	10.14 ± 4.67ab	10.81 ± 4.33ab
MC (%)	14.55 ± 1.85ab	17.11 ± 2.18c	14.66 ± 2.80ab	12.84 ± 1.87a	13.22 ± 2.47a	16.10 ± 1.95bc	14.53 ± 2.17ab	14.25 ± 2.40a	13.58 ± 2.37a
EC (μs·cm^−1^)	43.50 ± 16.27a	56.09 ± 12.49b	57.99 ± 10.46b	60.18 ± 12.35b	58.11 ± 11.10b	44.54 ± 8.49a	36.70 ± 9.32a	36.91 ± 11.59a	35.71 ± 10.28a
BD (g·cm^−3^)	1.23 ± 0.12bc	1.09 ± 0.20a	1.24 ± 0.18bc	1.39 ± 0.15d	1.42 ± 0.16d	1.18 ± 0.09ab	1.16 ± 0.16ab	1.24 ± 0.12bc	1.34 ± 0.14cd
TPO (%)	54.43 ± 4.24cd	57.91 ± 6.16d	54.65 ± 6.37cd	50.05 ± 4.06ab	49.11 ± 4.62a	53.74 ± 2.41bc	53.93 ± 4.61c	52.51 ± 3.92abc	51.93 ± 4.73abc
SMBP (mg·kg^−1^)	6.23 ± 0.88c	6.42 ± 1.14c	5.51 ± 1.10ab	5.38 ± 0.62ab	4.90 ± 0.71a	6.23 ± 0.87c	5.89 ± 1.01bc	5.49 ± 0.79ab	5.15 ± 0.73a
SMBC (mg·kg^−1^)	182.79 ± 22.69b	218.95 ± 56.67c	179.94 ± 59.09b	153.92 ± 21.16ab	135.75 ± 27.17a	216.55 ± 61.71c	179.02 ± 62.28b	165.46 ± 36.45ab	147.51 ± 30.92ab
SMBN (mg·kg^−1^)	68.89 ± 1.87ab	71.39 ± 3.22b	68.75 ± 2.75ab	63.04 ± 2.38ab	58.99 ± 2.23a	68.76 ± 2.42ab	61.85 ± 2.03ab	59.29 ± 2.01a	58.31 ± 1.94a
S-ACP (U·g^−1^)	48.64 ± 9.16a	53.57 ± 13.11a	50.37 ± 11.79a	47.92 ± 11.89a	49.13 ± 10.99a	51.22 ± 8.36a	49.24 ± 9.92a	47.35 ± 8.99a	46.96 ± 10.22a
S-UE (U·g^−1^)	14.28 ± 2.43bcd	16.81 ± 2.86 e	15.79 ± 2.30d	14.41 ± 1.51bcd	14.96 ± 2.18cd	14.91 ± 2.43cd	13.44 ± 1.53abc	13.02 ± 1.37ab	12.23 ± 2.48a
S-SC (U·g^−1^)	2.18 ± 0.32bc	2.41 ± 0.34d	2.03 ± 0.26ab	1.95 ± 0.19a	1.86 ± 0.14a	2.40 ± 0.45d	2.28 ± 0.37cd	1.94 ± 0.20a	1.99 ± 0.14ab
S-β-GC (U·g^−1^)	56.14 ± 8.56ab	57.74 ± 8.31b	55.39 ± 8.30ab	53.85 ± 6.17ab	50.03 ± 6.73a	56.02 ± 9.21ab	54.88 ± 7.91ab	50.76 ± 8.95a	51.24 ± 7.12ab
T-Pro (U·g^−1^)	7.46 ± 1.75ab	9.16 ± 1.77c	8.54 ± 1.59bc	8.53 ± 1.38bc	8.01 ± 1.10abc	8.23 ± 1.46bc	7.39 ± 1.33ab	7.50 ± 1.71ab	6.96 ± 1.29a

There are significant differences in the one-way ANOVA of various compound patterns with different letters (LSD, p < 0.05).

The soil N, P, and K contents of the upper soil layer (0–10 cm) were the richest, while the soil microbial and enzyme activities were also the most active, and significantly higher than in the 11–20 cm and 21–30 cm layers (p < 0.05). However, there were no significant differences between the MC soil layers (Table 2). In all soil layers, the coefficients of variation for the TN, TP, SOC, AN, AK, AP, SMBC, and EC exceeded 20%, with the results showing that soil quality indicators had strong spatial variations under different management intensities and durations.

**Table 2:** Changes in soil chemical, physical, and biological indicators of *C. dabieshanensis* forests between different soil layers.

Indicator	0–10 cm		11–20 cm		21–30 cm	
	Mean ± standard deviation	CV (%)	Mean ± standard deviation	CV (%)	Mean ± standard deviation	CV (%)
TN (g·kg^−1^)	2.96 ± 0.67c	22.66	2.19 ± 0.58b	26.46	1.81 ± 0.46a	25.73
TP (g·kg^−1^)	0.52 ± 0.16c	31.14	0.35 ± 0.12a	33.44	0.43 ± 0.13b	28.96
TK (g·kg^−1^)	23.13 ± 3.75b	16.23	20.22 ± 3.00a	14.84	20.63 ± 3.96a	19.20
pH	5.06 ± 0.18a	3.56	5.11 ± 0.15a	2.86	5.21 ± 0.16b	3.15
SOC (g·kg^−1^)	23.92 ± 5.69c	23.78	16.01 ± 3.42b	21.40	12.89 ± 3.08a	23.90
AN (mg·kg^−1^)	299.84 ± 56.59c	18.87	215.99 ± 47.23b	21.86	174.36 ± 43.16a	24.75
AK (mg·kg^−1^)	71.46 ± 15.57c	21.78	51.80 ± 9.47b	18.29	46.77 ± 10.21a	21.83
AP (mg·kg^−1^)	17.02 ± 3.66b	21.52	8.97 ± 1.96a	21.88	8.31 ± 2.98a	35.90
MC (%)	15.05 ± 2.50a	16.65	14.31 ± 2.35a	16.41	14.26 ± 2.70a	18.94
EC (μs·cm^−1^)	59.63 ± 13.51b	22.66	44.33 ± 10.86a	24.50	39.29 ± 11.95a	30.40
BD (g·cm^−3^)	1.14 ± 0.17a	15.10	1.27 ± 0.16b	12.71	1.35 ± 0.13c	9.77
TPO (%)	56.48 ± 4.99c	8.84	52.66 ± 4.81b	9.13	50.27 ± 3.76a	7.48
SMBP (mg·kg^−1^)	6.29 ± 0.92c	14.60	5.48 ± 0.90b	15.36	4.94 ± 0.64a	12.89
SMBC (mg·kg^−1^)	210.90 ± 55.48c	26.31	174.16 ± 38.51b	22.11	141.57 ± 32.74a	23.12
SMBN (mg·kg^−1^)	77.61 ± 10.49c	13.51	66.52 ± 7.13b	10.71	48.96 ± 3.40a	6.94
S-ACP (U·g^−1^)	61.92 ± 6.04b	9.76	43.02 ± 5.59a	12.99	43.20 ± 4.91a	11.37
S-UE (U·g^−1^)	16.18 ± 2.55c	15.76	14.34 ± 2.20b	15.37	12.76 ± 1.29a	10.10
S-SC (U·g^−1^)	2.30 ± 0.41c	17.88	2.12 ± 0.28b	13.27	1.92 ± 0.18a	9.16
S-β-GC (U·g^−1^)	60.38 ± 6.50c	10.76	53.81 ± 6.42b	11.94	47.83 ± 6.19a	12.93
T-Pro (U·g^−1^)	9.41 ± 1.27c	13.46	7.85 ± 1.30b	16.53	6.67 ± 0.81a	12.10

There were significant differences in the one-way ANOVA of various compound patterns with different letters (LSD, p < 0.05).

### Establishment of minimum data set for soil quality evaluation indicators

**Figure 2:**
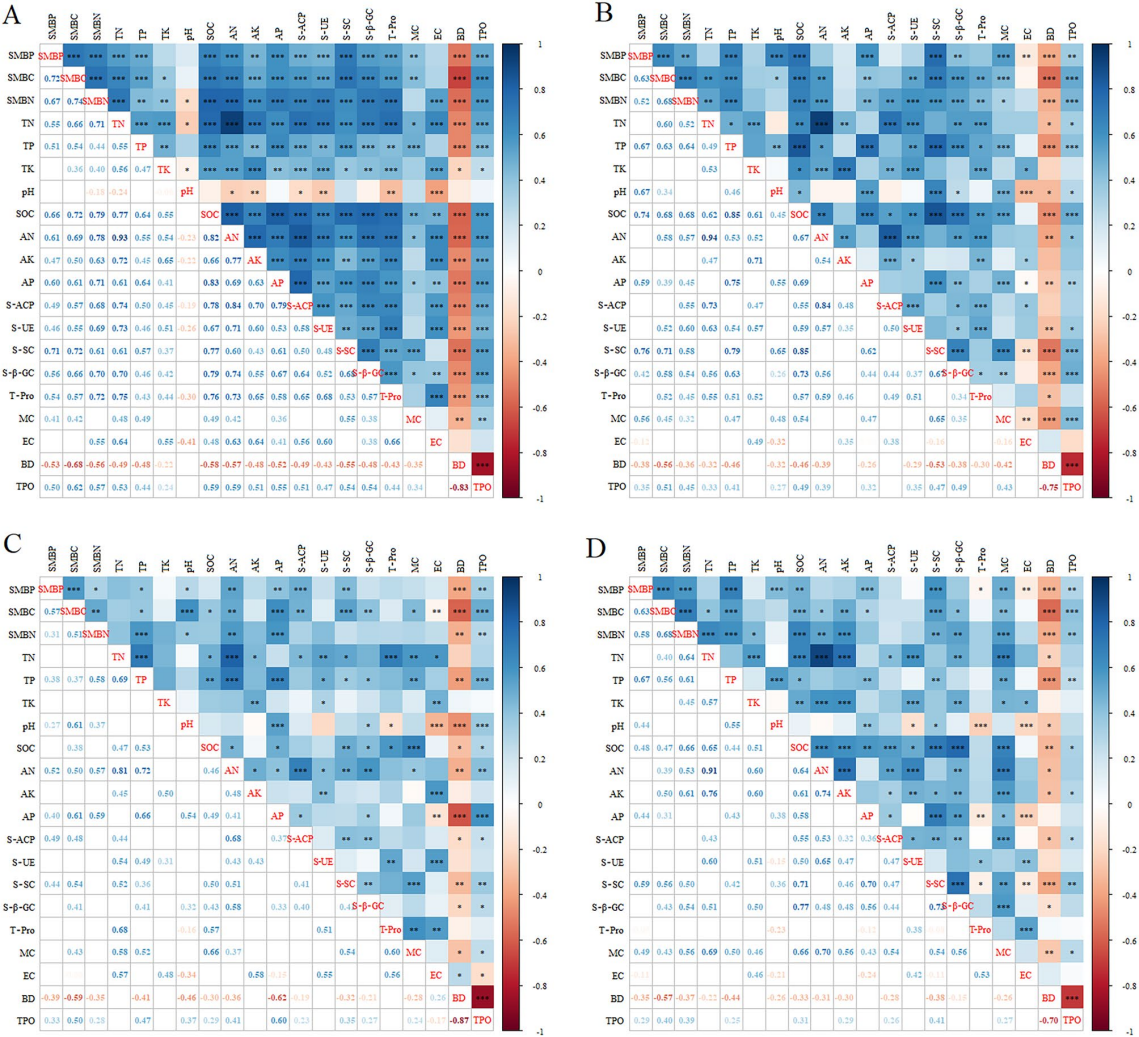
In all soil layers (**A**), 0–10 cm soil layer (**B**), 11–20 cm soil layer (**C**), 21–30 cm soil layer (**D**), soil quality evaluation indicator correlation. * Significance of p < 0.05. ** Significance of p < 0.01. ***Significance of p < 0.001.

Principal component analysis was performed on all soil indicators in the three soil layers (Table 3). The results revealed that the principal components of three eigenvalues > 1 explained 73.06% of the impacts of soil property indicators on soil quality in the study area, with each group explaining 30.46%, 29.82%, and 12.78%, respectively. The correlation analysis results of soil quality evaluation indicators implied that there was a strong correlation between the indicators with high Norm values (Fig. 2). According to the screening principle, pH, AN, and SMBN with high Norm values were selected to enter the MDS.

**Table 3:** Principal component analyses results of soil quality indicators.

Indicator	Principal component					MDS_1_	MDS_2_	MDS_3_	MDS	OMDS
	0–30 cm					0–10 cm	11–20 cm	21–30 cm	0–30 cm	0–30 cm
	PC1	PC2	PC3	Common factor variance	Norm					
TN	0.75	0.467	0.232	0.835	2.206					
TP	0.317	0.358	0.691	0.706	1.612		Yes	Yes		Yes
TK	0.698	− 0.027	0.471	0.709	1.880	Yes				
pH	− 0.55	0.081	0.677	0.768	1.747				Yes	
SOC	0.589	0.627	0.353	0.864	2.185	Yes		Yes		Yes
AN	0.729	0.564	0.178	0.882	2.285	Yes	Yes	Yes	Yes	Yes
AK	0.74	0.365	0.145	0.703	2.047					
AP	0.472	0.592	0.329	0.681	1.928					
MC	0.161	0.253	0.696	0.574	1.333					
EC	0.878	0.041	− 0.025	0.774	2.171		Yes			
BD	− 0.095	− 0.841	− 0.135	0.735	2.078		Yes	Yes		Yes
TPO	0.151	0.824	0.097	0.711	2.052	Yes				
SMBP	0.235	0.686	0.334	0.637	1.851					
SMBC	0.289	0.767	0.293	0.758	2.059					
SMBN	0.57	0.686	0.044	0.797	2.189			Yes	Yes	
S-ACP	0.658	0.509	0.139	0.711	2.057					
S-UE	0.69	0.413	0.073	0.652	1.983					
S-SC	0.234	0.654	0.509	0.741	1.883					
S-β-GC	0.464	0.614	0.239	0.649	1.924					
T-Pro	0.72	0.448	0.065	0.723	2.089		Yes			
Eigenvalue contribution of variance (%)	30.46	29.82	12.78	–	–					
Cumulative rate of accumulated variance (%)	30.46	60.28	73.06	–	–

Subsequently, principal component analyses of the properties of the three soil layers were performed. For the 0–10 cm soil layer, four indicators (TK, SOC, AN, and TPO) were ultimately selected to enter the MDS_1_. For the 11–20 cm soil layer, five indicators (TP, AN, EC, BD, and T-Pro) were selected to enter the MDS_2_. For the 21–30 cm soil layer, five indicators (TP, SOC, AN, BD, and SMBN) were ultimately selected to enter the MDS_3_. The TP, SOC, AN, and BD were chosen to construct the OMDS as they served as cross-common metrics in the MDS_1_, MDS_2_, and MDS_3_ (Table 3). The filtration rates of soil indicators in the MDS and OMDS were 85% and 80%, respectively, which reduced the influence of redundant data on the soil quality evaluation.

### Determination of the weight of comprehensive soil quality evaluation indicators

Principal component analyses of MDS and OMDS indicators were conducted, and the weights of the indicators were calculated according to the common factor variance of each indicator (Table 4). The weights of pH, AN, and SMBN in the MDS were 0.100, 0.457, and 0.444, respectively, while those of TP, SOC, AN, and BD in the OMDS were 0.219, 0.295, 0.275, and 0.211, respectively. Further, the weights of the SOC and AN were higher in the two datasets, which suggested that the SOC and AN were the most important indicators for the evaluating of soil quality in the study area.

**Table 4:** Weight values of soil quality indicators in the TDS, MDS, and OMDS.

Indicators	TDS	MDS	OMDS
TN	0.057		
TP	0.048		0.219
TK	0.049		
pH	0.053	0.099	
SOC	0.059		0.295
AN	0.060	0.457	0.275
AK	0.048		
AP	0.047		
MC	0.039		
EC	0.053		
BD	0.050		0.211
TPO	0.049		
SMBP	0.044		
SMBC	0.052		
SMBN	0.055	0.444	
S-ACP	0.049		
S-UE	0.045		
S-SC	0.051		
S-β-GC	0.044		
T-Pro	0.049		

### Comprehensive evaluation of SQI

**Figure 3:**
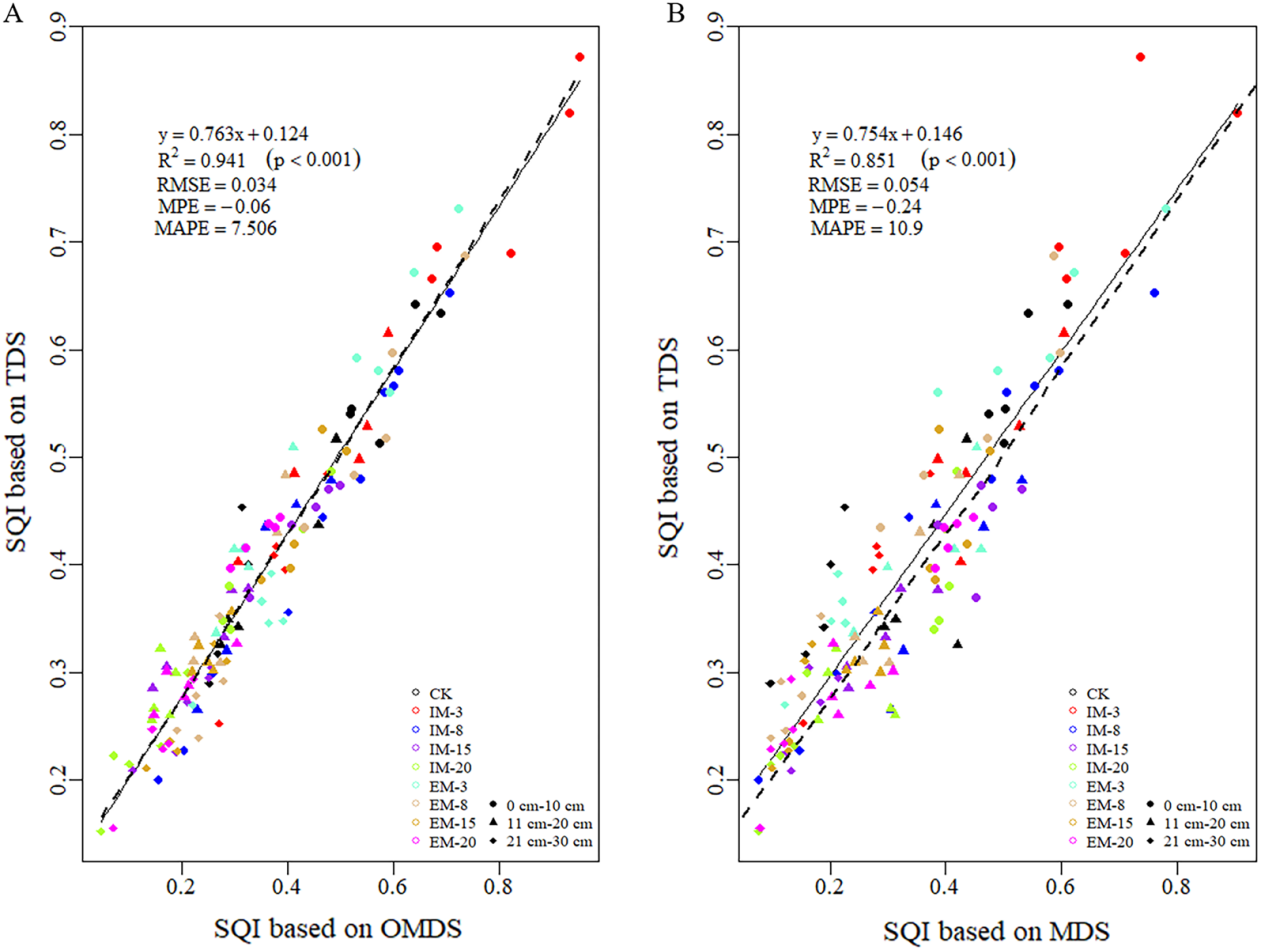
Linear relationships between SQI-TDS and SQI-OMDS (**A**), SQI-TDS and SQI-MDS (**B**). *RMSE* root mean squared error, *MPE* mean predictive error, *MAPE* mean absolute percentage error.

For this study, the TDS-SQI was linearly fitted and had a significant linear relationship with OMDS-SQI and MDS-SQI (P < 0.001), (Fig. 3), and the R^2^ of the linear fitting equation were 0.941 and 0.851, respectively. It was shown that the indicators screened by OMDS and MDS had potent representative effects on the evaluation of soil quality in *C. dabieshanensis* forests, and the OMDS dataset improved the prediction accuracy of soil quality in the study area. Furthermore, it could more accurately reflect the soil quality, as it tolerated less error (RMSE = 0.034, MPE = − 0.06, MAPE = 7.506).

**Figure 4:**
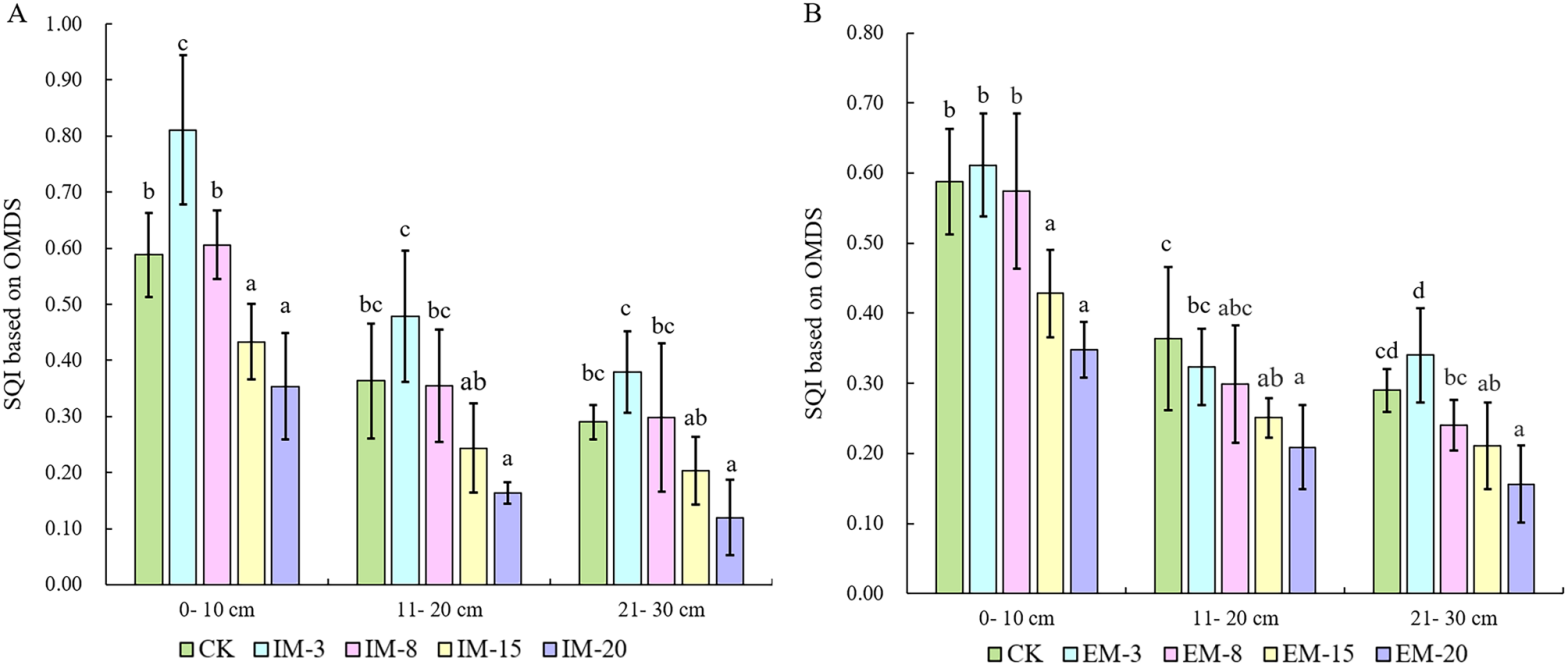
Soil quality index (SQI) of intensive management (IM) and extensive management (EM) *C. dabieshanensis* forests. There were significant differences in the one-way ANOVA of various compound patterns with different letters (LSD, p < 0.05), and the significance signs are independent for each soil depth.

OMDS was employed to comprehensively evaluate the soil quality of *C. dabieshanensis* forests under different management intensities and durations (Fig. 4). The results indicated that the soil quality in the early stage of management (IM-3, EM-3) was significantly improved compared with that of forest that were unmanaged (CK). Among all forest stands, IM-3 had the highest SQI, which was 0.81 ± 0.13, 0.47 ± 0.11, and 0.38 ± 0.07, respectively. For IM-20, the SQI of each soil layer was the lowest soil quality at 0.35 ± 0.09, 0.16 ± 0.02, and 0.12 ± 0.06, respectively. With longer management durations the soil quality gradually declined, and it was worth noting that the rate of soil quality degradation under intensive management was higher than that of extensive management. The changes in soil quality in the soil layers were essentially the same, where the soil quality decreased significantly in the deeper soil layers.

## Discussion

### Changes in soil attributes of *C. dabieshanensis* forests under different management intensities and durations

The anthropogenic management of forests is the most common interference, as it can have positive or negative effects on stand productivity and soil fertility contingent on the intensity and duration of management^5,9,34^. Fertilization and the removal of understory vegetation in subtropical forests with limited soil fertility can rapidly increase the productivity of target trees over the short term^10^. In this study, the TN, AN, AK, and SMBC contents of the soil increased rapidly, while the activities of urease, sucrase, and protease were higher than those in the unmanaged (CK) stands (Table 1). The early application of fertilizers supplemented the N, P, and K contents of the soil; accelerated the growth of plants; promoted the reproduction of soil microorganisms; and secreted more soil enzymes for nutrient conversion^35^. Further, a large quantity of plant residues was retained in situ following the removal of understory vegetation, and the increase in humus further promoted the accumulation of soil SOC and N. A recent study revealed that increased SOC promoted N chelation, which was verified in this study through the strong positive correlation between SOC and N^2,9^.

It was observed that with longer management duration, the nutrient content, soil biomass, soil enzyme activity, and soil pH steadily decreased. In particular, the soil TP, SOC, AN, AK, and AP contents decreased significantly. Numerous studies have shown that the long-term application of fast-acting N-based fertilizers can significantly alter the soil chemical properties, which results in nutrient imbalances by reducing the soil pH, and modifying the composition of soil buffer systems^36–38^. A study by Qiao^39^ concluded that the long-term application of N or N-based compound fertilizers led to the accumulation of nitrate in the soil, which aggravates the loss of abundant alkali cations through leaching, weakened its buffering capacity, and led to acidification. Soil microorganisms are critical for the decomposition of soil organic matter, where the biological enzymes secreted by microorganisms and plant roots are essential catalysts that induce the transformation of organic matter to inorganic compounds^10,40^. It is believed that soil microorganisms are more suited for weakly acidic environments in contrast to those that are weakly alkaline. A lower soil pH impacts the populations and activities of soil microorganisms, reduces the secretion of enzymes, as well as the decomposition rates and nutrient turnover capacities of organic matter^41,42^. A study by Tu^43^ concluded that the long-term application of fast-acting N-based fertilizers reduced the availability of P, and led to its restriction. The increased application of fertilizers stimulates plant growth, while also increasing their demand for P, which translates to the depletion of soil phosphate reservoirs if P is not added in time^44^. The removal of vegetation exacerbates soil erosion in woodlands, which may be another important driver for the reduction of soil P^9,26^. Furthermore, it is believed that the long-term application of chemical fertilizers reduces the organic matter content of soil, destroys its aggregate structures, and reduces porosity that leads to compaction^45^. Soil compaction further boosts nitrogen loss via nitrate leaching, which is primarily affected by decreased surface evaporation and soil water holding capacities^46^. In this study, we found a significantly negative correlation between the BD and SOC, observing that the BD increased and TPO decreased (both significantly) under prolonged management (Fig. 2).

There were significant differences in the soil chemical, physical, and biological indicators between the different soil layers in *C. dabieshanensis* forests (P < 0.05). Typically, the N, P, K, and SOC levels were highest in the topsoil, which decreased with soil depth as did the soil enzyme activities, microbial mass, TPO, and EC (Table 2). This may have been due to fertilization, which directly increased the content of numerous elements in the topsoil. Plant residues that remained following the removal of vegetation also accumulated in the surface layer, which increased the content of organic matter and stimulated microbial activities. The higher availability of organic matter may be of benefit toward increasing the functional diversity of soil microorganisms, while the cyclic transformation of C and N is promoted^9,47^. In deeper soil layers litter and plant residues become scarce and the leaching of inorganic salts gradually decreases; thus, soil nutrients are typically reduced with soil depth^48^. The root systems of herbs and shrubs are primarily distributed through shallow soil, where the interspersion of plant roots is an important factor in improving air permeability^24^. Unlike other indicators, the soil pH increases in deeper soil layers and the degree of acidification is low. Fertilization and land use have been identified as the main causes of soil acidification^49^, the long-term application of N-based fertilizers leads to increased net outflows of nitrate via soil leaching and surface runoff, which exacerbates soil acidification^12^. Furthermore, the removal of understory vegetation reduces species richness and cover, aggravates surface soil erosion, and induces the loss of copious salt-based ions (Ca^2+^, Mg^2+^, K^+^, Na^+^), which is another essential factor that cannot be ignored^12,26^.

### Evaluation of soil quality in *C. dabieshanensis* forests under different management intensities and durations

The evaluation of soil quality is an important aspect of forest management, which can assist managers with the planning of adjustments over time to improve productivity^18,24^. In this study a MDS (AN, SMBN, and pH) was established by reducing the dimensionality of the principal components of 20 soil indicators. Further, the influence of soil layers on soil quality was considered to optimize the selection of MDS indicators to establish OMDS (TP, SOC, AN, and BD). The linear fitting results between SQI-TDS and SQI-OMDS, SQI-TDS and SQI-MDS showed that OMDS could more objectively and accurately reflect the soil quality status of the study area (Fig. 3). Several researchers have generated statistics based on the selection of soil evaluation indicators. Their results suggested that the SOC, pH, AP, AN, EC, BD, and SMBC were high-frequency indicators of the MDS^18^, which was relatively consistent with the indicators selected for this study.

The results of the SQI score revealed that compared with unmanaged (CK) forests, the soil quality of early management forests (IM-3, EM-3) was improved, with the soil quality steadily decreasing under prolonged management. The qualities of the soil between different layers were generally manifest as the surface layer (0–10 cm > middle layer (11–20 cm) > lower layer (21–30 cm), which aligned with the temporal and spatial variations in soil nutrients^10,19^. Nutrient loss and increased soil erosion were the direct causes of soil quality degradation. In this study, with TP, SOC, AN, and BD being the main factors that affected the soil quality of *C. dabieshanensis* forests. Combined with the temporal and spatial changes of these four indicators, and compared with the data of other regions^50^, the main issues in current *C. dabieshanensis* forests were determined to be the lack of organic matter, soil compaction, and long-term intensive management, which may still have certain P restrictions. It is generally believed that the application of organic fertilizer, or the addition of carbon biomass are effective means for improving soil fertility and alleviating acidification^51^. Organic fertilizer contains nutrients that are required by plant microorganisms, which not only promotes plant growth but also creates a more suitable environment for microbial reproduction, and accelerates the conversion rate of nutrients in the soil. Biochar possesses a large specific surface area and porous structure, which can improve the air permeability and water retention capacities of soil and enhance its structure^52–54^. In addition, the proper retention of shrubs and herbs is necessary, as multiple layers of canopy and constant ground cover can increase stormwater interception and reduce surface runoff, which can reduce nutrient loss^24^. Abundant fine roots can reduce nutrient loss by driving soil aggregation and capturing leached nutrients from deep soil layers^2,55^.

## Conclusion

For this study, the soil quality of *C. dabieshanensis* forests under different management intensities and durations was evaluated based on a minimum data set (MDS) and an optimized minimum data set (OMDS).

1. The results revealed that early intensive management can significantly improve soil quality; however, prolonged intensive management leads to soil degradation, and intensive management is more likely to lead to soil degradation than extensive management. This verified that our first and second hypotheses were correct.
2. The soil TP, SOC, AN content, and BD in *C. dabieshanensis* forests are the key factors that affect soil quality, which deserve more attention for long-term observations. This was not quite consistent with our third hypothesis (pH is not the most critical factor that affects soil quality in *C. dabieshanensis* forests).
3. Based on the spatiotemporal variations in soil indicators and SQI, we suggest that the frequency of understory vegetation removal should be reduced, and organic rather than chemical fertilizers should be used. The results of this study are significant for guiding the sustainable management and soil restoration of *C. dabieshanensis* forests.

Finally, we recommend stopping the removal of understory vegetation; retaining certain herbs and shrubs; increasing the amount of P-rich organic fertilizer, reducing or stopping the application of chemical fertilizers; and adding a suitable quantity of organic carbon to maintain the appropriate balance of C, N, and P in the soil. These steps might facilitate the alleviation of soil acidification in forests that have been intensively managed for more than 15 years.

## Data Availability

The datasets generated during and/or analyzed during the current study are available from the corresponding author upon reasonable request.
